# Variance characteristic preserving common spatial pattern for motor imagery BCI

**DOI:** 10.3389/fnhum.2023.1243750

**Published:** 2023-11-09

**Authors:** Wei Liang, Jing Jin, Ren Xu, Xingyu Wang, Andrzej Cichocki

**Affiliations:** ^1^Key Laboratory of Smart Manufacturing in Energy Chemical Process, Ministry of Education, East China University of Science and Technology, Shanghai, China; ^2^Shenzhen Research Institute of East China University of Science and Technology, Shenzhen, China; ^3^g.tec Medical Engineering GmbH, Schiedlberg, Austria; ^4^Systems Research Institute of Polish Academy of Science, Warsaw, Poland; ^5^Department of Informatics, Nicolaus Copernicus University, Toruń, Poland

**Keywords:** motor imagery, EEG, brain-computer interface, common spatial pattern, variance characteristic preserving

## Abstract

**Introduction:**

The common spatial patterns (CSP) algorithm is the most popular technique for extracting electroencephalogram (EEG) features in motor imagery based brain-computer interface (BCI) systems. CSP algorithm embeds the dimensionality of multichannel EEG data to extract features of motor imagery tasks. Most previous studies focused on the optimization of the time domain and the spectrum domain of EEG signal to improve the effectiveness of CSP, whereas ignoring the constraint on the projected feature space.

**Methods:**

This study proposed a variance characteristic preserving CSP (VPCSP) that is modified by a regularization item based on graph theory. Specifically, we calculated the loss of abnormalities of the projected data while preserving the variance characteristic locally. Then the loss could be rewritten as a matrix with the introduction of the Laplace matrix, which turned it into a generalized eigenvalue problem equivalent to CSP. This study evaluated the proposed method on two public EEG datasets from the BCI competition. The modified method could extract robust and distinguishable features that provided higher classification performance. Experimental results showed that the proposed regularization improved the effectiveness of CSP significantly and achieved superior performance compared with reported modified CSP algorithms significantly.

**Results:**

The classification accuracy of the proposed method achieved 87.88 %, 90.07 %, and 76.06 % on public dataset IV part I, III part IVa and the self-collected dataset, respectively. Comparative experiments are conducted on two public datasets and one self-collected dataset. Results showed that the proposed method outperformed the reported algorithm.

**Discussion:**

The proposed method can extract robust features to increase the performance of BCI systems. And the proposal still has expandability. These results show that our proposal is a promising candidate for the performance improvement of MI-BCI.

## 1. Introduction

Brain-computer interfaces (BCIs) are communication systems that decode the information from the brain to control external devices (Romero-Laiseca et al., 2020; Zhang et al., 2021). Among the tasks for generating inputs for BCI systems, motor imagery (MI) is the mental imagination of movement without muscle’s activity, which depends on the users’ mental imagination of body movement without muscle activity (Aggarwal and Chugh, 2019). There is a variety of non-invasive and invasive ways of recording brain activities. For instance, Electroencephalogram (EEG), functional magnetic resonance imaging (fMRI), electrocorticography (ECoG), and magnetoencephalography (MEG) have been used as the input signal of BCI systems (Weiskopf et al., 2004; Rouse et al., 2016; Corsi et al., 2019). Of these acquisition ways, EEG is widely applied in BCI research, since it provides a low-cost and non-invasive way for bioelectric signal acquisition.

During the motor imagery tasks, the event-related desynchronization (ERD) and event-related synchronization (ERS) phenomena can be observed in EEG signals (Pfurtscheller and Neuper, 1997). Motor imagery related area presents regular spectrum changes within the alpha frequency band and beta frequency band, which provides the primary for distinguishing the motor imagery tasks. In practice, the capacity to recognize the EEG signals that correspond to the motor imagery tasks is essential for the reliability and effectiveness of MI-based BCI systems. Since EEG-based acquisition for motor imagery tasks has limitations such as low signal-to-noise ratio (SNR) and artifacts, decoding the motor imagery from EEG is challenging (Kevric and Subasi, 2017; McFarland and Wolpaw, 2017). Various algorithms have been proposed for feature extraction of EEG signals, in order to obtain high classification performance for BCI systems (Jafarifarmand et al., 2018; Chaudhary et al., 2020; Sadiq et al., 2020).

Of these algorithms, common spatial pattern (CSP) has been widely studied in feature extraction for motor imagery task classification. CSP algorithm was proposed to extract ERD/ERS features by projecting the multi-channel EEG signals (Müller-Gerking et al., 1999; Sun et al., 2020). The dimension of the channels is reduced by a spatial filter, which maximizes the variance of one class while minimizing the variance of the other one. This algorithm is an efficient tool to extract features for classifying motor imagery EEG signals (Meng et al., 2009; Xie et al., 2017).

However, the performance of CSP algorithm is affected by various factors such as the outliers (Qi et al., 2015). Since the CSP algorithm processes the covariance, the outliers may bring a negative impact on the spatial filter and then reduce the classification performance. To solve this problem, researchers have expanded CSP in many enhanced variants. Lotte and Guan (2011) proposed a theoretical framework for regularized CSP (RCSP) algorithm. Lemm et al. (2005) developed the common spatio-spectral patterns (CSSP) algorithm using the time delay embedding method, allowing for individual tuning of the temporal filter parameters at each EEG channel. To extend the temporal filter, Dornhege et al. (2006) introduced the finite impulse response (FIR) filter into CSP and proposed a common sparse spectral spatial pattern (CSSSP). Frequency band also has an impact on the effectiveness of the CSP method. Novi et al. (2007) proposed sub-band CSP (SBCSP), which used multiple frequency bands to extract CSP features and enhanced the classification performance. But for multi-frequency bands, not all frequency bands are conducive to the classification performance for the reason that some frequency bands contain little information on motor imagery. To increase the efficacy of CSP features, the filter bank CSP (FBCSP) chose discriminative filter bands with feature selection techniques (Ang et al., 2008). Considering the spatial and temporal domain, Qi et al. (2015) developed a novel framework for the CSP algorithm to optimize the spatial and temporal filters. Mishuhina and Jiang (2018) utilized a feature weighting and regularization method to use all CSP filters instead of using several CSP filters, which enhanced the classification accuracy. Combining temporal filters and spatial filters, Jiang et al. (2020) proposed an efficient CSP algorithm to alleviate the overfitting problem.

Most previous studies focused on the optimization of the time domain and the spectrum domain. These methods ignored the robustness of the projected space. In this study, we focused on the projected space of the CSP algorithm and modified the CSP algorithm to enhance the effectiveness of the CSP feature. Since the CSP algorithm reduced the dimensionality of spatiality but did not reckon the robustness of the resulted space, the outliers in the time domain could easily affect the extracted features and cause misclassification of the classifier. We proposed a new version of CSP with smoothing regularization. Considering the smoothness of the projected space, a regularization was developed and added to the CSP algorithm, which aimed at stabilizing the projected space and reducing the influence of outliers.

The rest part of the paper is organized as follows. Section 2 introduces our method. Section 3 presents the experimental results and the details of our proposed framework. Then the experimental results are discussed in Section 4. Section 5 presents the conclusion of this study.

## 2. Methods

### 2.1. Common spatial pattern

In terms of feature extraction of motor imagery BCI systems, the CSP methods have been applied and extended widely in numbers of researches. The CSP algorithm builds a spatial filter $w\in R^{C}$ for multi-channel EEG data, which aims to find projections that maximize the separation of two classes (Ramoser et al., 2000). In detail, the spatial filter is generated by maximizing the variance/power of one class while minimizing the variance/power of another class in resulted space (Noh and de Sa, 2013).

The multi-channel EEG data is denoted as $X_{i}\in R^{C\times T}$, where i indicates the $i^{th}$ sample, C is the number of channels, T is the number of sample points in time series and $\epsilon_{n}$ is the $n^{th}$ class set. The covariance of the EEG data can be estimated as:


(1) $$\Gamma_{n}=\frac{1}{\left|\epsilon_{n}\right|}\sum_{i\in \epsilon_{n}}X_{i}X_{i}^{⊤}$$

Spatial filters of CSP are defined as $W=\left[w_{1},w_{2},\ldots ,w_{2K}\right],W\in R^{C\times 2K}$, where K denotes the employment of K pairs of filters. And the embedding feature of $n^{th}$ class EEG data is denoted as $z_{n}=X^{⊤}w$. Then the objective function of CSP can be formulated as follows:


(2) $$\begin{array}{rr} w_{\text{opt}} & =\arg\max_{w}\frac{{z_{1}}^{⊤}z_{1}}{{z_{2}}^{⊤}z_{2}}\\ \; & =\arg\max_{w}\frac{w^{⊤}\Gamma_{1}w}{w^{⊤}\Gamma_{2}w} \end{array}$$

The above problem can be solved by the Lagrange multiplier method with equality constraint: $w^{⊤}\Gamma_{2}w=1$, and $\Gamma_{n}$ is symmetric positive definite. Then we can solve the generalized eigenvalue problem to obtain the spatial filer w:


(3) $$\Gamma_{2}^{-1}\Gamma_{1}w=\lambda w$$

Hence, 2K eigenvectors corresponding to the K smallest and the K largest eigenvalues of $\Gamma_{2}^{-1}\Gamma_{1}$, are obtained as the spatial filters $W=\left[w_{1},w_{2},\ldots ,w_{2K}\right]$, resulting in $Z=X^{⊤}W$. The feature vector of $k^{th}$ filter is transformed by logarithmic transformation for classification:


(4) $$f_{k}=\log\left(\frac{\mathrm{var}\left(Z_{k}\right)}{\sum_{i=1}^{2K}\mathrm{var}\left(Z_{i}\right)}\right)$$

where $Z_{j}$ is the $j^{th}$ column of the matrix Z.

### 2.2. Variance characteristics preserving spatial pattern

The feature extraction algorithm described above obtains efficient features for recognition in motor imagery-based BCI systems. However, before the logarithmic transformation, the feature vectors z are easily affected by abnormal points. In this study, we aim to mitigate the effect of this type of point to improve the robustness of the CSP algorithm. Therefore, we consider z as a graph, with building connections at l points per interval.

For the embedded vector $z\in R^{T}$, reducing the loss of two connected points can $G=\left(V,E\right)$ preserve local variance characteristics while declining the sequence’s abnormalities. $V=\left\{v_{1},v_{2},\ldots ,v_{n}\right\}$ denotes the nodes of the graph, and $E=\left\{e_{1},e_{2},\ldots ,e_{m}\right\}$ denotes the edges of the graph. The adjacency matrix of the graph is defined as:


(5) $$A_{i,j}=\left\{\begin{array}{ll} 1 & \text{if}\;|i-j|=l\\ 0 & \text{othe}r\text{wise} \end{array}\right.$$

where $A\in R^{T\times T}$, $A_{i,j}$ denotes the elements of A, l is a user-defined parameter, $l\in \left\{1,\ldots ,T\right\}$.

**Figure 1:**
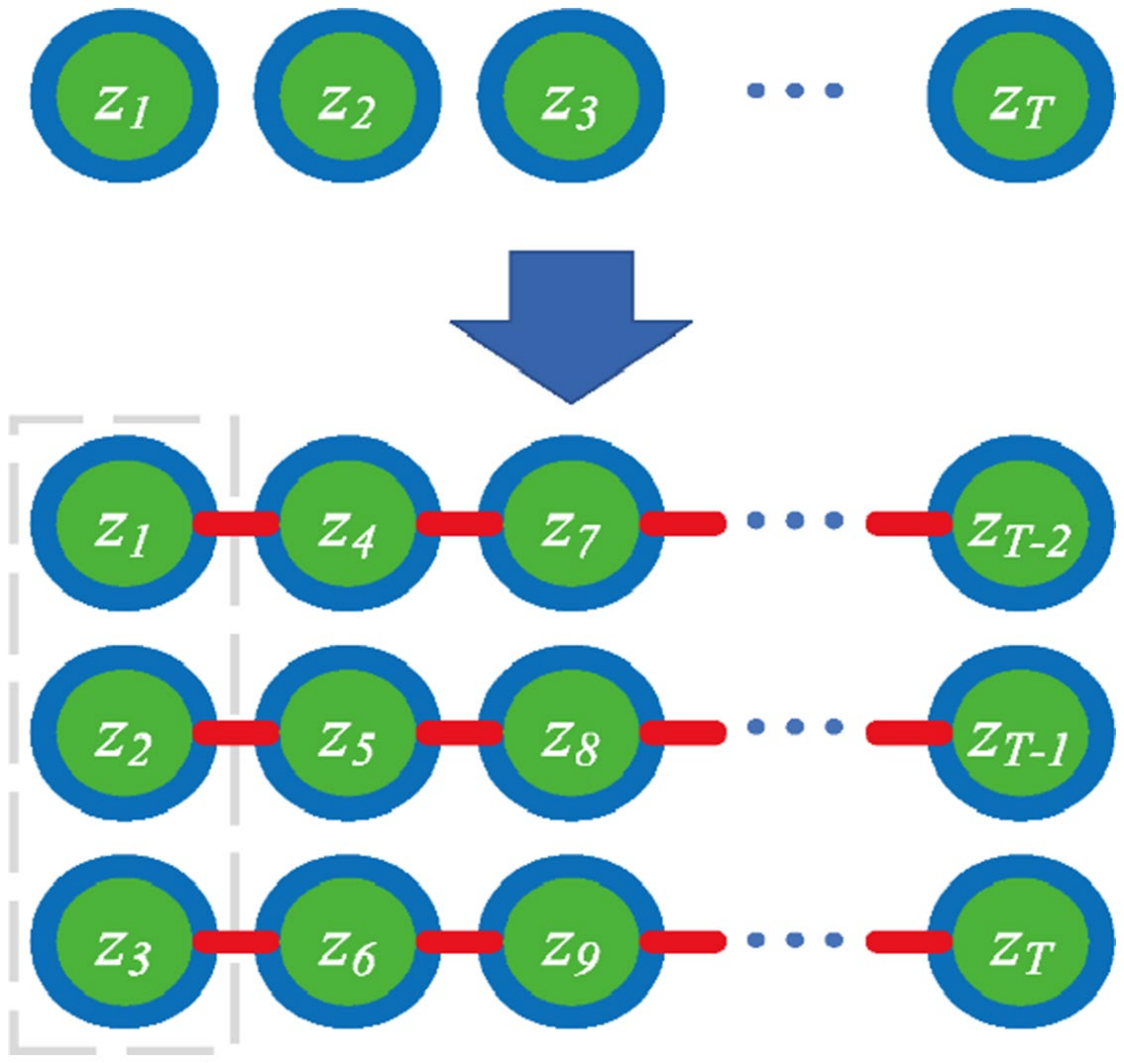
Illustration of the designed graph for projected space.

Figure 1 shows an example (l=3), we try to reduce the abnormalities of the whole sequence (as the difference between connected points with red lines), whereas preserving the local variance characteristic (as the gray box in Figure 1).

In this study, the embedded feature z can be viewed as a sequence that is generated from the channel dimension by a spatial filter. The regularization term is designed to be the sum of the Euclidean distances of any two points in the graph. We quantified the loss as Eq. (6). The loss function is designed to calculate the difference between two nodes separated by l points (satisfying the condition |i−j|=l). However, when |i−j|<l, the internal inter-node difference is not calculated, so the internal variance information is preserved as the loss function decreases. This allows the subsequent processing of the filtered feature to retain discernible energy differences, so we call it variance characteristics preserving CSP (VPCSP). With the decrease of the loss function, the difference between each interval l point of the filtered feature decreases, which makes the energy information of the filtered feature more stable.


(6) $$\begin{array}{cc} R\left(z\right) & =\sum_{i,j}{\left(z_{i}-z_{j}\right)}^{2}A_{i,j}\\ \; & =\sum_{i,j}\left(z_{i}^{2}+z_{j}^{2}-2z_{i}z_{j}\right)A_{i,j}\\ \; & =\sum_{i}z_{i}^{2}D_{i,i}+\sum_{j}z_{j}^{2}D_{j,j}-2\sum_{i,j}z_{i}z_{j}A_{i,j}\\ \; & =2z^{⊤}\left(D-A\right)z\\ \; & =2z^{⊤}Lz \end{array}$$

where $z_{i}$ is the $i^{th}$ of the embedded vector z, L is the Laplacian matrix, such that L=D−A, and D is the degree matrix.


(7) $$D_{i,j}=\left\{ \begin{array}{ll} \deg\left(v_{i}\right) & \text{if}\;i=j\\ 0 & \text{otherwise} \end{array}\right.$$

where $\deg\left(v_{i}\right)$ denotes the number of edges connecting to node $v_{i}$. In Eq. (6), $R\left(z\right)$ represents the degree of smoothness of the projected features. Therefore, minimizing Eq. (6) can make the projected data smooth. As can be seen in Eq. (5), due to several adjacent points are not connected, the local variance characteristics are not weakened as the features become smooth. The proposed item tries to decline the outliers which could make an impact on the CSP features as Eq. (4).

In terms of the spatial filter, the proposed method needs to optimize two goals. Like the CSP algorithm, VPCSP tries to find spatial filters that maximize the projected power of one class of data while minimizing the projected power of another class of data. In addition, VPCSP also tries to minimize the proposed regularization item. The above two terms can be written as the objective function in the form of a generalized Rayleigh quotient.


(8) $$\begin{array}{rr} J\left(w\right) & =\frac{w^{⊤}\Gamma_{1}w}{\left(1-\beta \right)w^{⊤}\Gamma_{2}w+\beta z_{1,2}^{⊤}Lz_{1,2}},\\ z_{1,2}^{⊤}Lz_{1,2} & =w^{⊤}X_{1}LX_{1}^{⊤}w+w^{⊤}X_{2}LX_{2}^{⊤}w \end{array}$$

where $\beta \in \left(0,1\right)$ is a user-defined parameter. When β=0, the above equation is equivalent to the traditional CSP.

Since $\Gamma_{1,2}$ and $X_{i}LX_{i}^{⊤}$ are symmetric positive definite matrices, we can simplify the above formula by the following abbreviations:


(9) $$M=\left(1-\beta \right)\Gamma_{2}+\beta \left(X_{1}LX_{1}^{⊤}+X_{2}LX_{2}^{⊤}\right)$$

Then we can give the objective function of the proposed VPCSP:


(10) $$\begin{array}{r} \max_{w}\frac{w^{⊤}\Gamma_{1}w}{w^{⊤}Mw}\\ s.t.w^{⊤}Mw=1 \end{array}$$

The solution to the above problem is the same as the traditional CSP algorithm since M is a symmetric positive definite matrix.


(11) $$M^{-1}\Gamma_{1}w=\lambda w$$

Further feature optimization is obtained using multiple constraints. Above we use one constraint item to optimize the projected feature. More constraint items can be used to obtain further optimization. In above Eq. (8), the constraint item can be replaced by the multiple constraint items as:


(12) $$L=L_{1}+L_{2}+L_{3}\ldots +L_{q}$$

where the $L_{i}$ denotes the Laplacian matrix that obtained with l=i in Eq. (5). In addition, to extract invariant features, we extended the observation signal by one delayed coordinate as Lemm et al. (2005): ${\hat{X}}^{\tau }=\left(\begin{array}{c} X\\ \delta X \end{array}\right)$, with this modification, the objective function described above could be rewritten by changing the covariance matrix.

**Figure 2:**
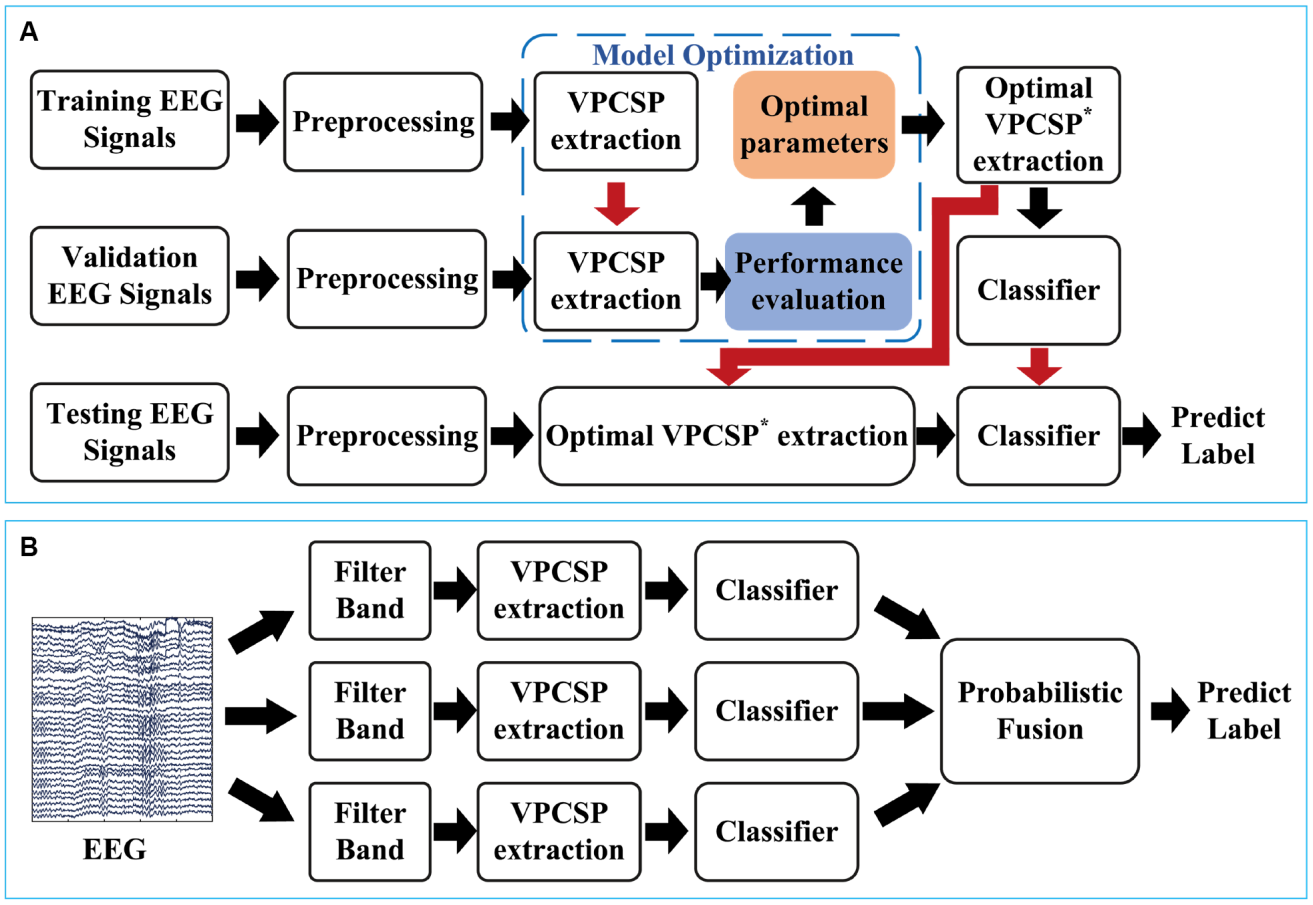
Illustration of the proposed framework. **(A)** The framework of the proposed optimization method for feature extraction. **(B)** The decoding procedure of the proposed method.

Additionally, the proposed method aimed at optimizing the extracted features from the spatial filters. However, most of the new algorithms that had been proposed recently used the filter bands technique, which achieved high classification performance on the MI-based BCI systems. Therefore, the combination of the spatial optimization and the spectra optimization would achieve further improvements. The EEG signals were divided into three frequency bands (4–20 Hz, 8–24 Hz, and 12–28 Hz), with 4th order Butterworth bandpass filters which covered alpha, beta, and mu rhythms. Since multiple frequency bands were used, multiple classifiers were generated. Probabilistic fusion was used to fuse the probabilistic score of classifiers from multiple branches, as in Figure 2B.

### 2.3. Bayesian optimization

In the proposed method, a number of user-defined parameters should be customized for each participant, including the l in Eq. (5), the β in Eq. (9) and the q in Eq. (12). To obtain the high performance of the proposed framework, Bayesian Optimization (BO) was introduced to optimize the user-defined parameters. BO works by constructing a posterior distribution of functions (Gaussian process) that best describes the function. The Expected Improvement (EI) was optimized in this work for parameters optimization (Bergstra et al., 2011).

In this study, we used Hyperopt on GitHub as the BO tool for optimization of the parameters in our experiments (Bergstra et al., 2013). Using five-fold split strategy, data was split into training set, validation set, and testing set (64, 16, and 20% respectively). Firstly, initial parameters (l, β, q) were used to train on the training set. Then, parameters were optimized by BO algorithm based on the performance on the validation set. This procedure contained fifty loops to optimize the parameters for the model. Finally, the optimized model was tested on the testing set as the evaluated performance in these experiments.

### 2.4. Modified CSP algorithms for comparison

In the space domain, the CSP algorithm had been modified with a number of methods to obtain an efficient spatial filter. Different spatial filters were created using different modification techniques. Therefore, we compared the performance of the proposed method and the CSP algorithm described below.

The Tikhonov regularization CSP (TRCSP) was introduced to the CSP algorithm for improving the performance (Lotte and Guan, 2011). The TRCSP aimed at mitigating the effect of artifacts and outliers. The Tikhonov regularization was considered as an effective regularization item in the proposed framework (Lotte and Guan, 2011).

The sparse common spatial pattern (SCSP) algorithm used $L_{1}$-norm regularization to modify the CSP algorithm (Yong et al., 2008). By introducing the regularization item designed, the sparse filter could be produce.

Lu et al. (2010) proposed the Regularized Covariance-Matrix Estimation (R-CSP) in small sample situations. R-CSP modified the covariance matrix by introducing a robust covariance matrix estimation technique.

The common spatio-spectral pattern (CSSP) modified CSP with the time delay embedding method (Lemm et al., 2005). The CSSP extended CSP algorithm to the state space, which aimed at obtaining invariant features.

The Filter Bank Common Spatial Pattern (FBCSP) (Ang et al., 2008): multiple bandpass filters were used to obtain CSP features in different frequency bands. Then, the mutual information based best individual feature algorithm was used to select the optimal filter bands with corresponding CSP features.

### 2.5. Classification

The support vector machine (SVM) has been widely applied in the BCI field (Miao et al., 2021). It is effective for the classification task on small dataset, such as motor imagery classification. SVM tries to find the separating the hyperplane with the maximum margin which makes the maximum distance between hyperplane and the nearest sample data (Cortes and Vapnik, 1995). It can be expressed as the following constrained optimization problem:


(14) $$\begin{array}{rr} \; & \min_{w,b,\zeta }=\frac{1}{2}w^{2}+C\sum_{i=1}^{N}\zeta_{i}\\ s.t. & y_{i}\left(w^{⊤}x_{i}+b\right)\geq 1-\zeta_{i},i=1,2,\ldots ,N\\ \; & \zeta_{i}\geq 0,i=1,2,\ldots ,N \end{array}$$

where $\zeta_{i}$ is the slack variable, $y_{i}$ is the true label of the data $x_{i}$.

In this study, we used the radial basis function kernel based SVM as the tool for classification (Amari and Wu, 1999).

## 3. Results

### 3.1. Dataset description

The effectiveness of the proposed VPCSP was verified in two datasets from public BCI competition datasets.

Dataset 1 was from the BCI Competition IV, part I (Zhang et al., 2012). The EEG data in this dataset consisted of 59 channels and were recorded at a sampling rate of 1,000 Hz. This dataset contained 7 participants’ EEG data. Since the 3 participants’ EEG data (‘c’, ‘d’, and ‘e’) were made artificially, we only consider the rest of the 4 participants for verification (‘a’, ‘b’, ‘f’, and ‘g’). Three motor imagery tasks (left hand, right hand, and foot imagery) were designed in the experiment, which used left, right and down arrows as cues separately for 4 s. Each subject performed only two tasks of them, a total of 200 trials. We used the data that was downsampled to 100 Hz. The following website had further information about this dataset: http://www.bbci.de/competition/iv/.

Dataset 2 was from the BCI Competition III, part IVa (Blankertz et al., 2006). This dataset consisted of 118-channel EEG data which were set as the 10–20 EEG system using 1,000 Hz sampling rate. In the experiment, the cues were displayed for 3.5 s in each trial and then participants relaxed in periods of random time length ranging from 1.75 to 2.25 s. Left hand, right hand, and right foot motor imagery tasks were set, but only two motor imagery tasks (right hand and right foot imagery tasks) were provided in this public dataset. It contained 5 healthy participants’ EEG data (marked: aa, al, av., aw, and ay) during the experiment and 280 trials total for each participant. We used the data that was downsampled to 100 Hz. The following website had further information about this dataset: http://www.bbci.de/competition/iii/.

Dataset 3 was collected in our lab. Its paradigm was similar to the public datasets from the BCI competition that used the left and right arrows as cues separately for left motor imagery task and right motor imagery task. In this experiment, the cures were displayed for three seconds. Dataset 3 consisted of 10 participants (S1,S2,…,S10) who were graduate students between the ages of 21 and 27 years. The experiments used 16-channel (FC5, FC1, FCZ, FC2, FC6, C5, C3, C1, CZ, C2, C4, C6, CP5, CP1, CP2, and CP6)with a sample rate of 600 Hz. A bandpass filter from 8 Hz to 32 Hz and a notch filter at 50 Hz was applied to remove artifacts. Each participant performs 120 trials of motor imagery tasks (60 trials per class).

### 3.2. Data preprocessing

The EEG signals were bandpass filtered from 8–32 Hz using the Butterworth filter (4th order). The Butterworth filter’s configuration aimed to eliminate high-frequency noise like power line noise while keeping track of the brain activity associated with motor imagery. In our experiment setup, we used all the EEG channels and the time window that covered the whole time after the cue except the first 0.5 s. In other words, the time windows used were [0.5, 3.5], [0.5, 3], and [0.5, 3] in Datasets 1, 2, and 3 separately.

### 3.3. Comparison results

We divided the dataset into three parts: training set, validation set, and testing set. Five-fold cross-validation was used to generate the test sets. The training process was applied in the training set, model selection was based on the validation set, and the test set was to estimate the performance of the algorithm. Since the proposed VPCSP method contains two parameters that need to be preset, we set a series of parameter subsets in the training fold. And then with the proposed model selection strategy, we evaluated all the parameter subsets and selected the optimal parameters in the training set and validation set. The framework of our study can be seen in Figure 2A.

To verify the effectiveness of the penalty item, we compared the performance of the proposed method and the traditional CSP algorithm firstly. In addition, most previous studies reckoned without the projected features. Instead, the EEG signal before projection was optimized mostly. Therefore, we compared the proposed VPCSP with the CSP algorithm improved by other modification methods in space domain.

Table 1 presented the performance comparison of various spatial filters, including two versions of the VPCSP algorithm (one without filter bands (FB) and the other with filter bands). Additionally, A Wilcoxon Signed Rank Test (Rey and Neuhäuser, 2011) was also used to assess the results. Between the compared methods and the proposed method, there are statistically significant differences between their classification performance (*p* < 0.05). With the same experimental condition (the same preprocessing method and the same classifier), only the feature extraction methods were different from each other. And the data met the requirements for statistical differences. It meant that the proposed method generated higher mean classification accuracy than the compared one.

**Table 1:** Comparison results with other modified CSP algorithms on two public datasets.

Subjects	Methods							
	CSP	SCSP	TRCSP	R-CSP	CSSP	FBCSP	VPCSP (w/o FB)	VPCSP (w/FB)
a	61.50	54.50	77.50	82.50	63.50	81.00	78.00	**85.00**
b	55.50	46.00	56.50	59.00	52.00	63.00	64.00	**77.00**
f	55.00	48.50	70.00	67.50	60.00	79.00	76.00	**93.50**
g	88.50	76.00	73.50	75.00	86.50	93.00	95.00	**96.00**
Mean	65.13	56.25	69.38	71.00	65.50	79.00	78.25	**87.88**
aa	72.86	80.71	69.64	71.79	75.00	72.14	76.79	**83.21**
al	94.29	96.07	89.29	83.21	96.07	94.64	98.21	**98.93**
av	55.36	46.07	58.21	61.79	61.43	68.21	65.71	**77.14**
aw	76.07	84.29	81.43	73.57	85.00	91.07	**97.14**	96.79
ay	86.07	85.00	86.90	86.07	92.50	91.79	90.36	**94.29**
Mean	76.93	78.43	77.09	75.29	82.00	83.57	85.64	**90.07**
Value of *p*	*<*0.05	*<*0.05	*<*0.05	*<*0.05	*<*0.05	*<*0.05	*<*0.05	*-*

The bold values denote the highest score achieved by corresponding methods.

Results showed that the VPCSP improved the performance of all subjects in all datasets compared with the traditional CSP algorithm. In two public datasets, the proposed method achieved the best performance in two datasets. Compared with the traditional CSP, average classification accuracy with VPCSP (w/o FB) increased over 13% on Dataset 1 and over 7% on Dataset 2, respectively. For subject ‘aw’, classification performance improved the most by approximately 14%. Furthermore, with the filter bands, the VPCSP achieved higher classification performance than that of VPCSP without FB. VPCSP with filter bands (VPCSP-FB) increased over 22% on Dataset 1 and increased over 13% on Dataset 2 comparing with the traditional CSP algorithm. The results demonstrated that the integration of the proposed spatial optimization and frequency domain optimization significantly enhanced the effectiveness of feature extraction for classification purposes. All comparison results showed statistical significance (*p* < 0.05).

To avoid the proposed model overfitting the public datasets, we collected the EEG data on MI task in our lab. The comparison result was presented on the Table 2. And this table presented the effectiveness of the proposed method. The proposed method achieved an average classification accuracy improvement of approximately 7% compared to the CSP method. Moreover, the robustness of the proposed method was also evident. As the results showed that the effectiveness of the feature extraction might fail in some cases compared to the baseline. Mostly, the proposed method showed a good robustness since only one subject failed in this experiment, which was superior to the reported methods.

**Table 2:** Comparison results with the reported methods on Dataset 3.

Methods	Subjects										
	S1	S2	S3	S4	S5	S6	S7	S8	S9	S10	Mean
CSP	58.89	65.13	56.67	64.44	74.44	84.44	55.56	81.11	**61.11**	90.00	69.18
R-CSP	56.67	63.85	58.89	64.44	68.89	78.89	43.33	76.67	54.44	85.56	65.16
TRCSP	54.44	57.05	57.78	60.00	68.89	70.00	50.00	81.11	60.00	91.11	65.04
CSSP	63.33	60.38	67.78	74.44	66.67	84.44	45.56	80.00	53.33	91.11	68.71
FBCSP	58.89	66.67	66.67	66.67	78.89	76.67	**63.33**	85.56	53.33	92.22	70.89
Proposed	**73.33**	**68.33**	**70.00**	**84.44**	**83.33**	**85.56**	56.67	**87.78**	58.89	**92.22**	**76.06**

The bold values denote the highest score achieved by corresponding methods.

Additionally, more evaluation indexes were used to evaluate the performance of the proposal. Recall measures the ability of a model to correctly identify all relevant instances. On the other hand, precision focuses on the accuracy of positive predictions made by a model. And accuracy, the widely used index in BCI, measures the overall correctness of predictions made by a model. In Table 3, the precision score, recall score and accuracy score were presented. Besides, a comparison with the recent approaches was also presented. In Table 4, our proposal achieved the highest average accuracy in the table.

**Table 3:** Evaluation of decoding model.

Subjects	Evaluation index		
	Precision	Recall	Accuracy
a	86.75	82.00	85.00
b	79.52	78.00	77.00
f	95.85	91.00	93.50
g	95.49	97.00	96.00
Mean	89.40	87.00	87.88
aa	83.80	87.14	83.21
al	98.67	99.29	98.93
av	79.51	75.71	77.14
aw	97.29	96.43	96.79
ay	95.95	92.86	94.29
Mean	91.04	90.29	90.07

**Table 4:** Classification accuracy of the proposed approach and existing approaches.

Study	aa	al	av	aw	ay	Average
Selim et al. (2017)	69.64	89.29	59.18	88.84	86.90	78.77
Dai et al. (2018)	68.10	93.88	68.47	90.58	84.65	81.14
Park and Chung (2018)	74.11	**100.00**	67.85	90.07	89.29	84.26
Selim et al. (2018)	**86.61**	**100.00**	66.84	90.63	80.95	85.00
Hou et al. (2022)	77.68	**100.00**	73.98	84.82	88.10	85.00
Proposed	83.21	98.93	**77.14**	**96.79**	**94.29**	**90.07**

The bold values denote the highest score achieved by corresponding methods.

### 3.4. Modified spatial filter

**Figure 3:**
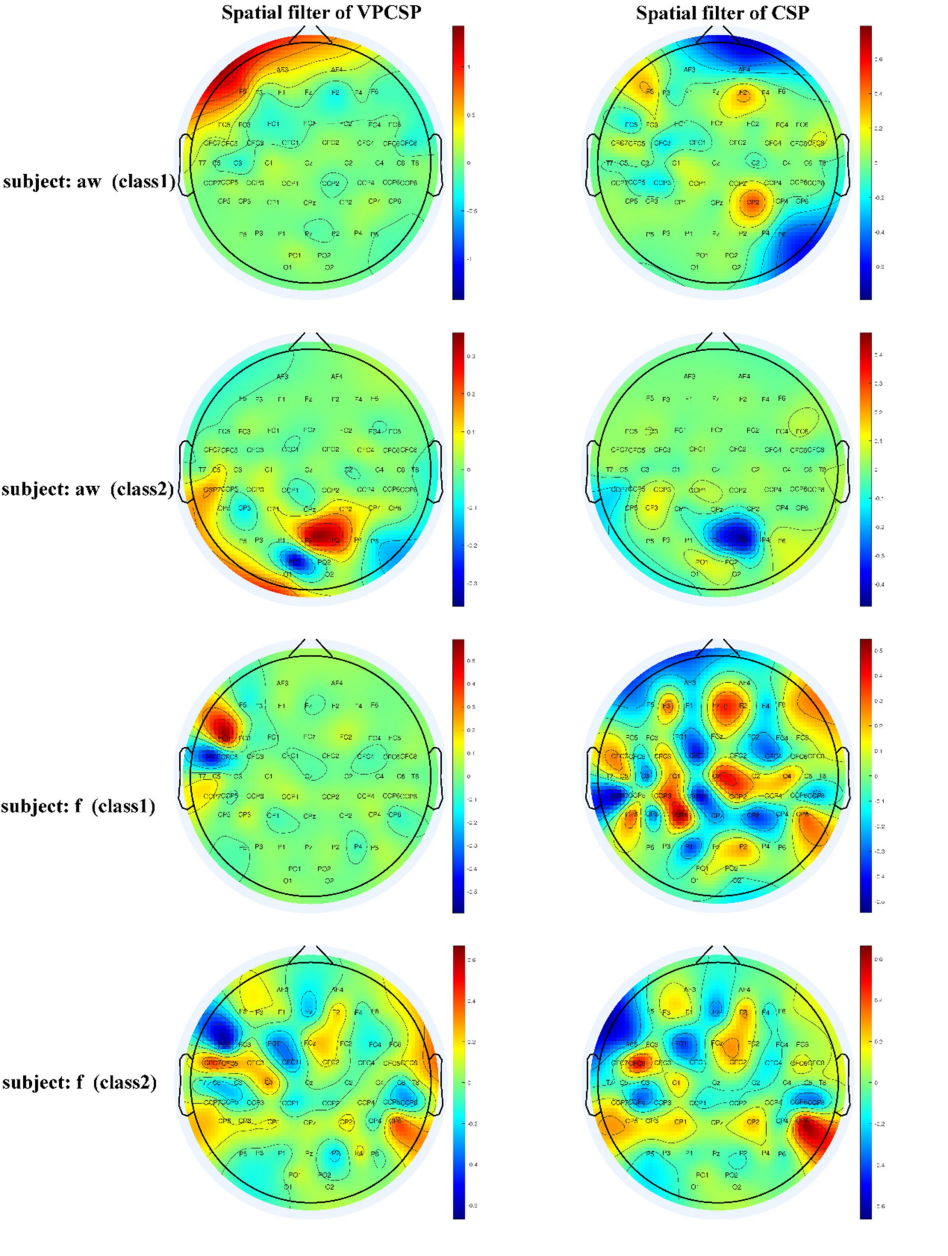
Spatial filter of proposed VPCSP and traditional CSP.

**Figure 4:**
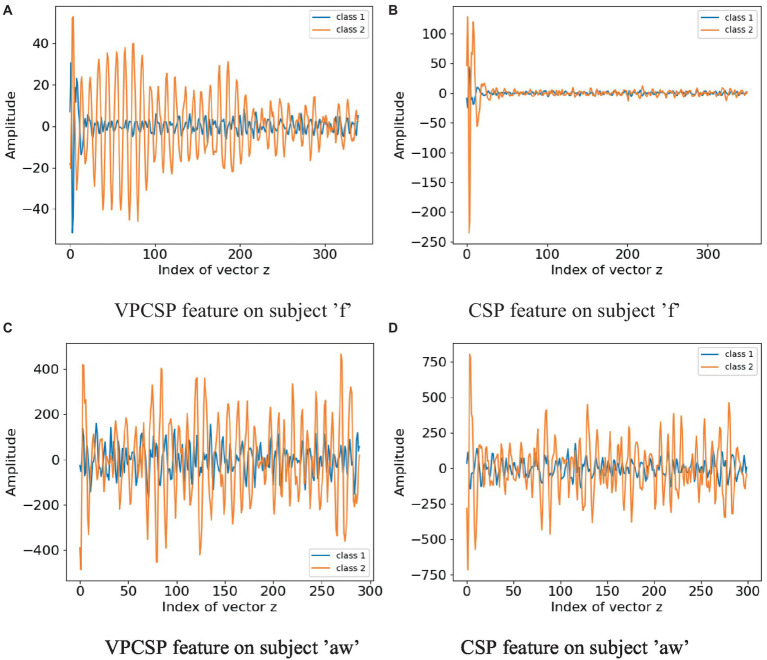
Electrode weight topography of spatial filters obtained by the proposed VPCSP and the traditional CSP algorithm. **(A)** VPCSP feature on subject ‘f’ **(B)** CSP feature on subject ‘f’ **(C)** VPCSP feature on subject ‘aw’ **(D)** CSP feature on subject ‘aw’.

The results show that the weights obtained by VPCSP (without filter bands) are smoother than those obtained by CSP. According to Figure 3, the spatial filter of VPCSP only adjusts the weights on a small number of leads, and the whole filter is smoother than that of CSP. At the same time, it can be seen that VPCSP generates several positive and negative spatial filters, such as Pz, P2 and PO1 in the figure. However, in combination with Figure 4, it is found that the feature amplitudes extracted by the two kinds of filters on aw are significantly different, especially in Class 2. It can be seen that the spatial filter of VPCSP in Class 2 realizes the elimination of outliers by weight compensation of Pz, P2 and PO1 (visible dark blue and dark red parts, representing larger negative and larger positive values respectively). It also shows that these outliers are distributed in the spatial domain. The same phenomenon can be found on subject f, and it is more obvious. In Figure 3, VPCSP smoothed the spatial filter of Class 1 more than CSP. In combination with Figure 4, it can be seen that in subject f, the absolute value of the amplitude extracted by the modified method is less than 50, and. The features extracted by CSP not only have large amplitudes but also have outliers. In other words, the differences in energy become more evident, and certain outliers are attenuated. Therefore, the proposed method not only makes the filtered feature have distinguishable variance characteristics but also generates filters that are robust to prevent artifacts and outliers.

### 3.5. Classification model comparison

The classification model utilized in this algorithm was a radial basis function kernel (RBF) based SVM classifier which was applied in the BCI field widely. Compared with the RBF-based SVM, the Linear Discriminant Analysis (LDA) (Fisher, 1936), which was also a standard classification model in the BCI field, was simpler. In certain scenarios, the classification performance relied on both the feature extraction module and the classification model. Hence, two standard classification models were employed and compared within the proposed feature extraction method, and the results are presented in Table 5. The comparison revealed that the two standard models attained comparable classification performance to the proposed feature extraction module (*p* > 0.05). As a result, the proposed method demonstrated similar effectiveness on both the SVM classifier and LDA classifier.

**Table 5:** Comparison results with different classification models.

Subjects	Models	
	SVM (RBF)	LDA
a	85.00	85.50
b	77.00	69.50
f	93.50	92.00
g	96.00	95.00
Mean	87.88	85.50
aa	83.21	84.64
al	98.93	99.29
av	77.14	73.21
aw	96.79	97.50
ay	94.29	95.71
Mean	90.07	90.07
*p*-value	-	>0.05

## 4. Discussion

### 4.1. Sensitivity analysis

**Figure 5:**
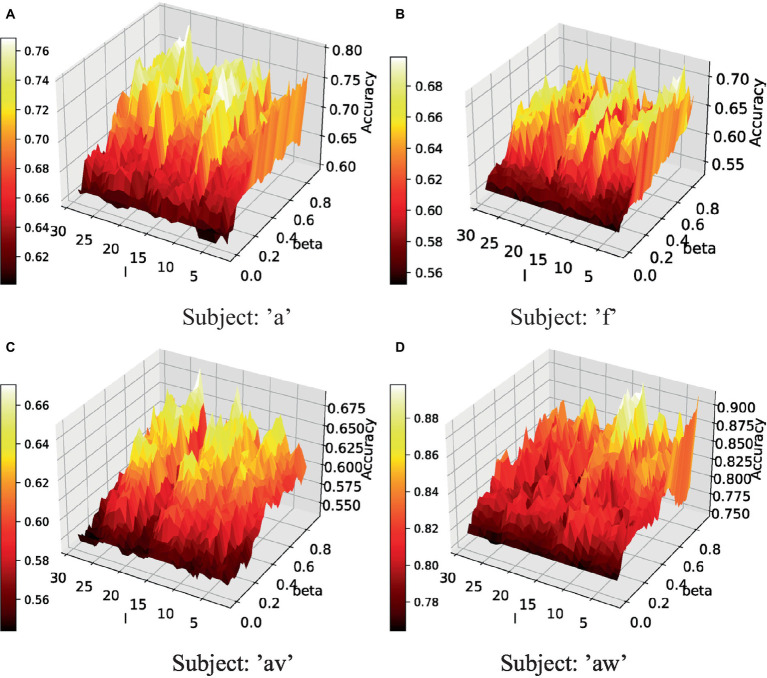
Parameter sensitivity of the proposed method in test fold [l: the parameter in Eq. (5), beta: the parameter β in Eq. (9)]. **(A)** Performance of subject ‘a’ under different parameters. **(B)** Performance of subject ‘f’ under different parameters. **(C)** Performance of subject ‘av’ under different parameters. **(D)** Performance of subject ‘aw’ under different parameters.

The proposed method contains two tunable parameters, including l in Eq. (5) and β in Eq. (8). The classification performance of four subjects under different parameter subsets is presented in Figure 5. When β=0, the proposed method is equal to the traditional CSP algorithm. Therefore, the performance of the CSP algorithm is shown by the black color in Figure 5. It shows that a large part of the parameter subsets can obtain good classification performance. Quite large parameter areas can obtain over 10\% improvement in accuracy. In this parameter range, the high classification performance obtained has little difference, which makes it easy to obtain optimal or suboptimal parameters.

Considering the classifier used in our method, several user-defined parameters probably had an impact on the classification performance. To show the influence of this factor, we analyzed two standard classification models with the same feature extraction procedure. The comparison of classification performance between the SVM classifier and the LDA is shown in Table 5. Statistical analysis was also done for these results, which suggested that the SVM classifier’s performance and that of the LDA classifier did not show a statistical difference (*p*  > 0.05). Therefore, the classifiers showed similar performance on the feature that was extracted by the proposed algorithm.

### 4.2. Computational efficiency

The computational efficiency directly affected the practical application of the proposed method. In our framework, Bayesian optimization required conducting iterations on the training set, which meant that our algorithm needed to consume more computing during the training process. Table 6 showed the computing time within one fold, including the training procedure and the testing procedure. The experiment ran on a personal computer with Intel(R) Xeon(R) Gold 5,222 3.80 GHz CPU and 128 GB of RAM. It indicated that the proposed framework consumed approximately 3 min in the training procedure, which was longer than that of the CSP method. Though consuming more time, it could meet the requirement of practical application and the classification accuracy obtained was much higher. The long computing time was cost in the training mode while the computing time in the testing mode was much shorter. It meant that our proposed method could output the command in a short time which met the requirement for practical application.

**Table 6:** Running time in one fold.

Methods	Running time	
	Train mode	Test mode
CSP	0.5360 s	0.0032 s
Ours	3.2107 min	0.0039 s

### 4.3. The characteristic of projected features

For the projected features, a comparison between the proposed VPCSP and the classical CSP algorithm on subjects ‘f’ and ‘aw’ was shown in Figure 4. The orange line denoted the average projected features of one class, whereas the blue line denoted the average features of the other one class. It could be observed that the projected features of VPCSP were more regular than that of CSP. In addition, the projected feature of VPCSP on subject ‘f’ was more discriminative compared with the classical CSP. From another point of view, the feature that was used to train the SVM classifier was calculated by Eq. (4), which contained the calculation of variance. Therefore, in terms of the power of the projected series of the proposed method and the CSP method, the proposed method could generate more robust power by calculating the feature in Eq. (4). In summary, in Figures 4A,B, it was shown that the features of VPCSP contained more discriminative variance characteristics than the classical CSP. In addition to the variance characteristics, as shown in Figure 4, the proposed method also mitigated the influence of the high amplitude artifacts. However, it is hard to conclude that the phenomena depicted in Figure 4 demonstrate consistency across the dataset since the associated visualizations may not reveal differences perceptible to the naked eye. We still can see that significant disparities can be observed in these two participants who exhibited substantial improvements.

### 4.4. Expandability and future work

The proposed VPCSP focused on the space domain of EEG data. We developed a regularization item for the CSP algorithm. The proposed method constrains the projected feature, which indicates that the spatial filters are constrained. From another point of view, the VPCSP without filter bands only improves the space domain of the CSP algorithm. And the time domain or the frequency domain of data is not considered in this scheme. Moreover, previous studies showed that the optimization of the time domain and frequency domain can improve the classification performance of the traditional CSP algorithm (Park and Chung, 2019; Zhang et al., 2019; Miao et al., 2021). Therefore, we considered the scheme that used the VPCSP with filter bands (VPCSP-FB). In this scheme, we utilize the filter bands technique to optimize the frequency domain of the extracted features further.

However, the proposed algorithm still has room for improvement. For instance, the time domain of this algorithm is still not optimized. And the frequency bands used in this research are limited. Considering more filter bands, effective feature selection methods could be utilized such as the least absolute shrinkage and selection operator (LASSO) method (Tibshirani, 1996). Thirdly, the classifier fusion strategy used in this work might be the easiest method. Other methods like the Fuzzy fusion method (Nazemi et al., 2017) could be considered in future work. Moreover, transfer learning has been applied in the BCI field (He and Wu, 2020). Consequently, the combination of these approaches and our method may generate new vitality in MI-based BCI field.

Actually, the state-of-the-art models mostly are the neural network models. But both the neural network models and the feature extraction models are evolving simultaneously. Although neural network models can achieve state-of-the-art performance, these models also have some defects in practical application. For one thing, these models tend to have higher complexity and more user-defined parameters. Compared with the CSP algorithm, training these models often requires more data to ensure the stability of the model. When the dataset is too small to train a large-scale CNN, there comes the overfitting problem that decreases the performance of CNN (Ma et al., 2022). This limits its promotion in practical applications, because each data sample requires users to perform tasks to obtain, users will feel tired because of the execution of tasks, so the amount of data is small. For another, some transfer learning algorithms have been successfully applied to CSP algorithms (He and Wu, 2020; Wu et al., 2022). Through these algorithms, subjects do not have to spend a lot of time on data collection to train the model, and only a small amount of data can be used to calibrate the criteria to obtain satisfactory classification performance, and then the BCI system can be used. This can improve users’ comfort with BCI systems to some extent. And the proposed method still has the enough expandability to these algorithms. Further studies are worth doing in future for this problem.

## 5. Conclusion

In this study, we propose a variance-feature-preserving CSP algorithm. We will focus on the characteristics of the projection space and develop a regularization project. We design a graph for the projected data and calculate the loss of the edges in the graph. Then the total loss of the projected data is taken as the regularization term. By introducing the designed regularization term, the local variance feature is preserved while solving the spatial filter, the deviation of the whole projection data is reduced, and the delay operator is combined for further optimization. In addition, we introduce Bayesian optimization algorithm to avoid manual selection of user-defined parameters and obtain excellent classification performance. The proposed feature extraction algorithm still retains scalability and can be further optimized with other transfer learning algorithms. The performance of the proposed model is verified on two public data sets and one self-collected data set. The experimental results show that compared with the existing improved CSP algorithm, the proposed method is more robust and can obtain better classification performance, which indicates that our method is an improvement of the CSP algorithm. It can be used for decoding of BCI system based on motor imagination tasks.

## Data availability statement

The raw data supporting the conclusions of this article will be made available by the authors, without undue reservation. Further inquiries can made to the corresponding author.

## Ethics statement

The studies involving humans were approved by Ethics Committee of East China University of Science and Technology. The studies were conducted in accordance with the local legislation and institutional requirements. The participants provided their written informed consent to participate in this study.

## Author contributions

WL raised the idea of the manuscript and designed the experiment. JJ made effective suggestions on the manuscript’s structure and provided the experimental site. RX has embellished the language of the manuscript and made key suggestions. XW and AC provided inputs for optimizing the data processing flow. All authors contributed to the manuscript revision, read, and approved the submitted version.
